# Supplementary material for: Super‐resolution imaging of proteins inside live mammalian cells with mLIVE‐PAINT
Source: Protein Sci. 2025 Jan 25;34(2):e70008. doi: 10.1002/pro.70008 (PMC11761688; doi:10.1002/pro.70008)
Supplement: Supplementary file 1 — Data S1. Supporting Information. [file PRO-34-e70008-s002.pdf]

Super-resolution imaging of proteins inside live mammalian cells with mLIVE-PAINT

Supplementary Information

Table of contents

1. Doxycycline induction response in mLIVE-PAINT cell line transfected with TOM20-101B
2. Distribution of localizations over time
3. Distribution of localization precisions
4. Distribution of residence times
5. Cluster detection and signal:noise estimation
6. Distribution of Nearest Neighbor (NN) distances

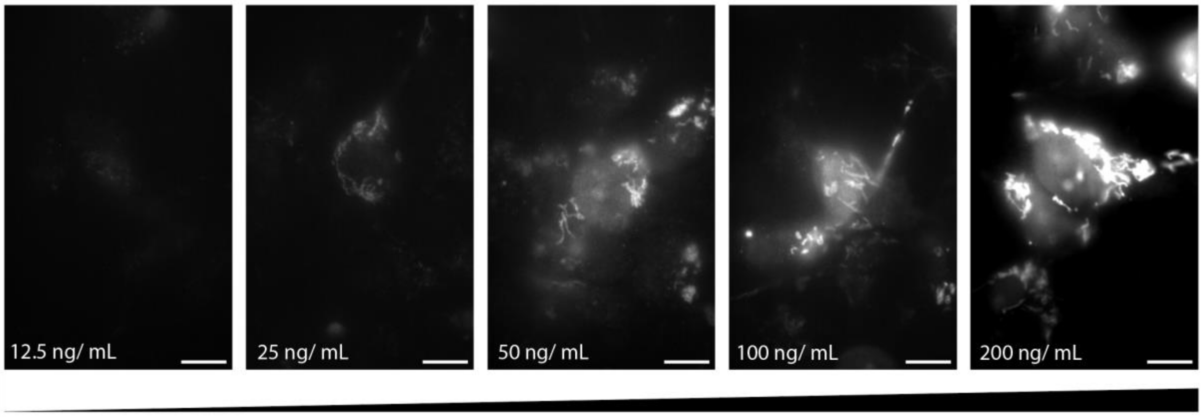

Figure 1: Doxycycline induction response in mLIVE-PAINT cell line transfected with TOM20-101B. Diffraction-limited images shown are time-integrated z-projections. At higher concentrations (200 ng/ mL) mitochondria are strongly labelled with the imager whereas minimal signal is detected at the low concentration (12.5 ng/ mL). Approximately 50 ng/ mL allowed sufficient localizations to be detected without excessive background. 10 μm scale bar.

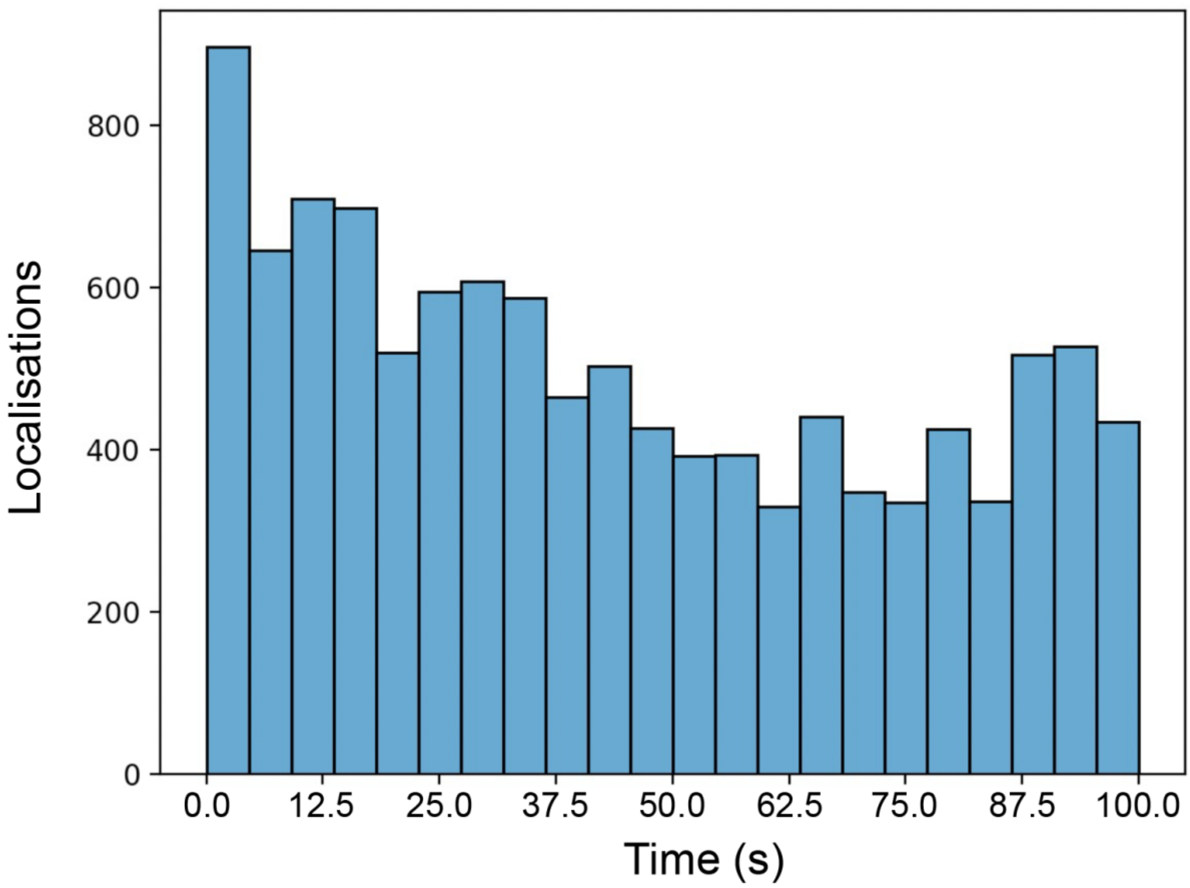

Figure 2: Distribution of detected localizations over a 100s imaging period when transfected with H2B-101B.

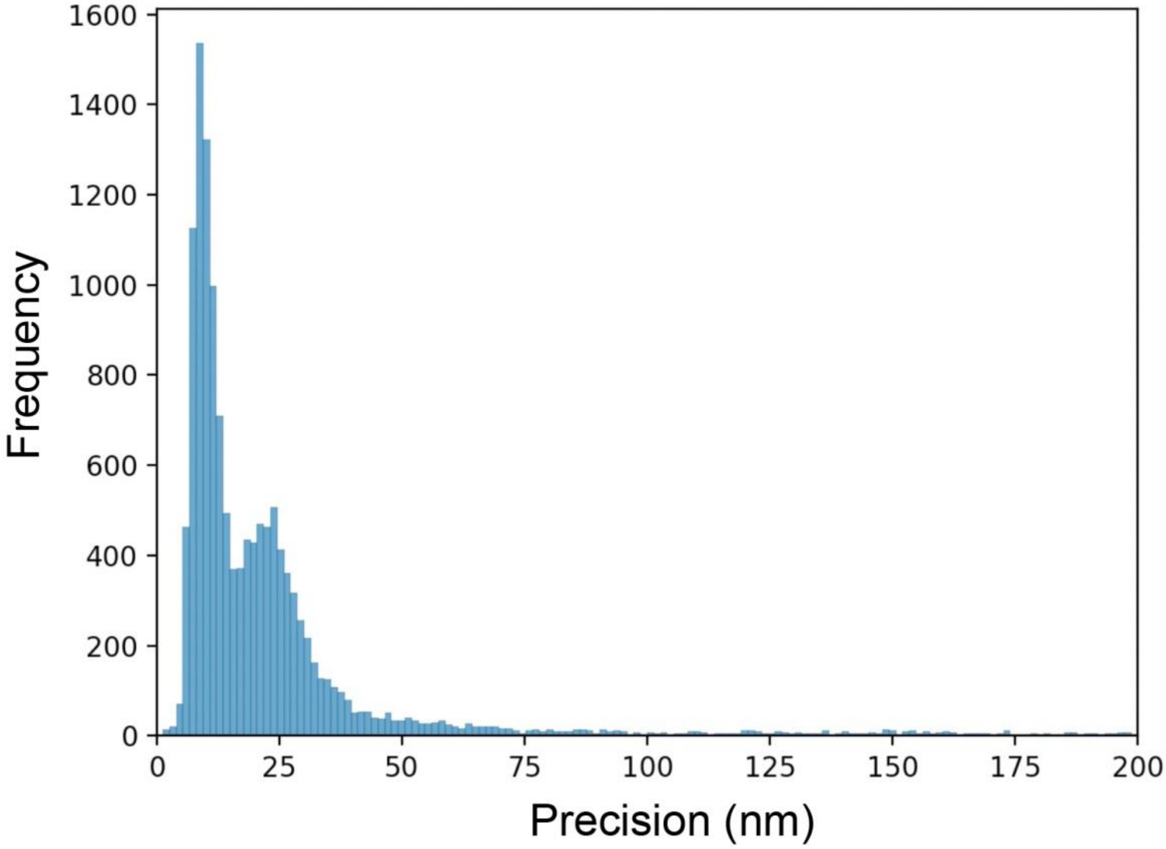

Figure 3: Distribution of precision values of detected localizations. A 30 nm threshold is set before SR image reconstruction.

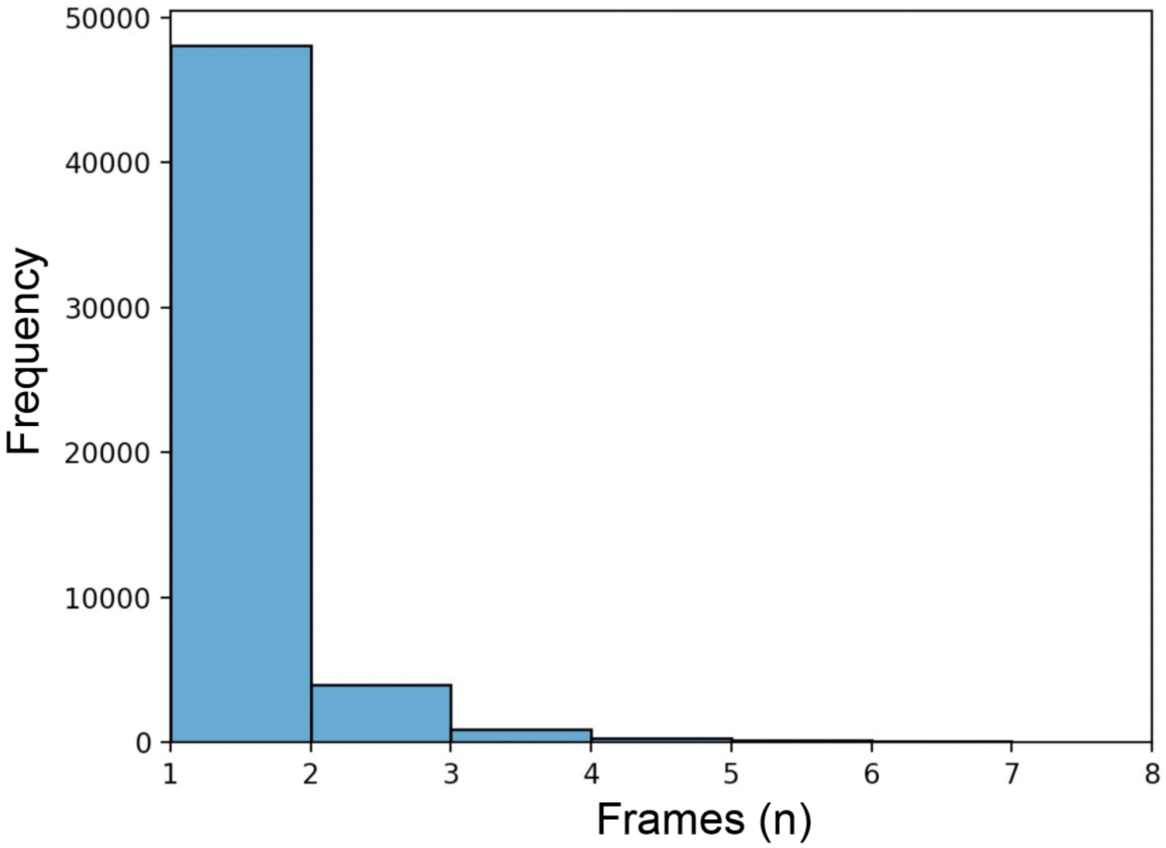

Figure 4: Distribution of residence times of detected localizations. The 'Merged' localizations function was used to link localizations across multiple frames. A large majority of localizations (>95%) last a single frame when imaged under 50 ms exposure.

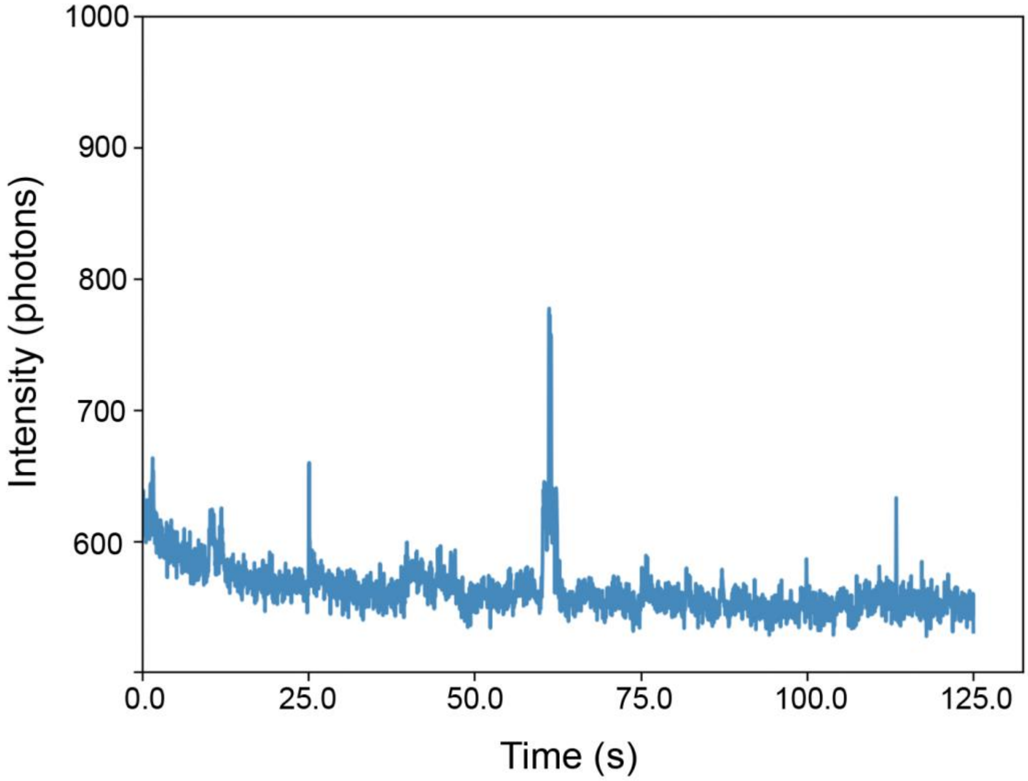

Figure 5: Intensity trace of a single spot ROI over a 125s imaging period. Spikes detected at the 25s and 115s timepoints show single imagers (mNG-101A) within the ROI while cumulative binding and unbinding of >1 imagers is seen at the 60s timepoint. The signal can also be inferred from this trace to be approximately 20% over the background.

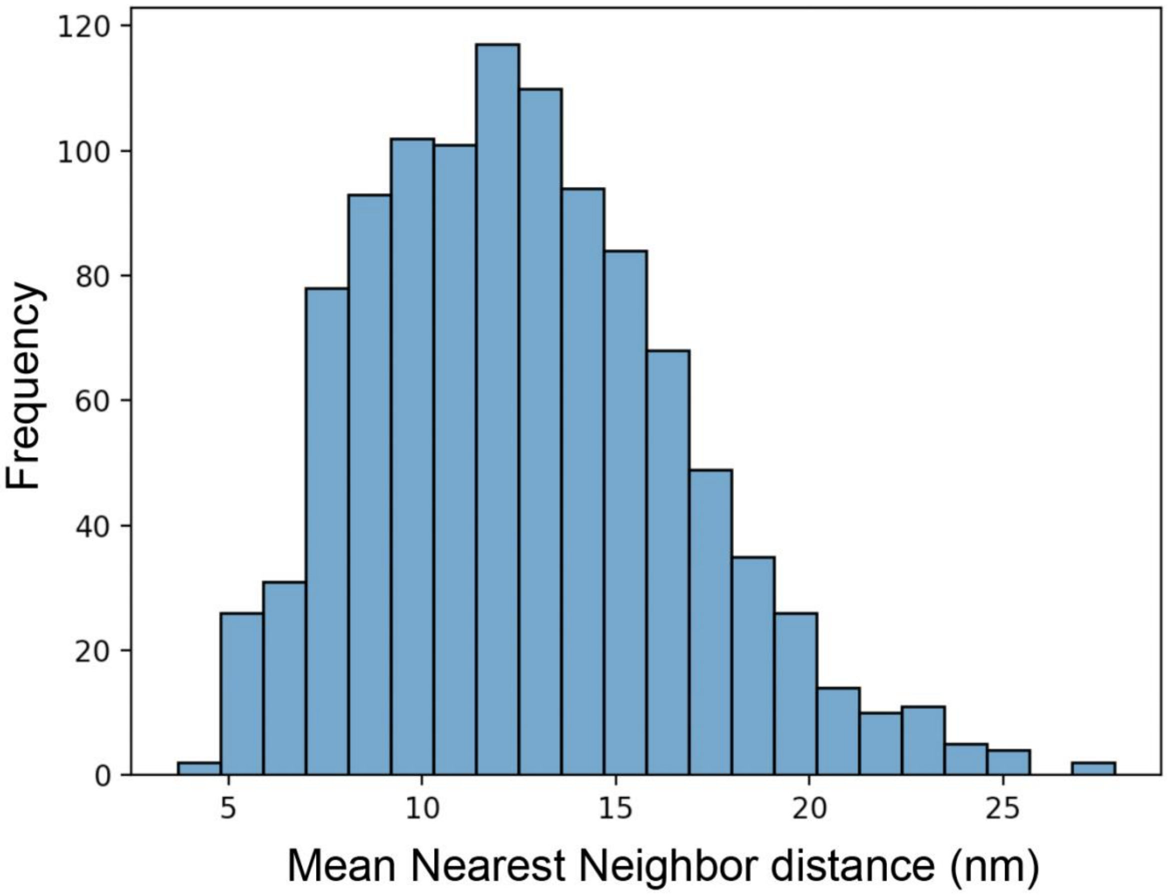

Figure 6: Distribution of mean nearest neighbor distances in a H2B-101B transfection scheme. Mean distances were calculated on a per-cluster basis after DBSCAN clustering.
